# Submaximal eccentric resistance training increases serial sarcomere number and improves dynamic muscle performance in old rats

**DOI:** 10.14814/phy2.70036

**Published:** 2024-10-03

**Authors:** Avery Hinks, Ethan Vlemmix, Geoffrey A. Power

**Affiliations:** ^1^ Department of Human Health and Nutritional Sciences, College of Biological Sciences University of Guelph Guelph Ontario Canada

**Keywords:** fisher 344/Brown Norway rats, muscle architecture, power, sarcomerogenesis, velocity

## Abstract

The age‐related loss of muscle mass is partly accounted for by the loss of sarcomeres in series, contributing to declines in muscle mechanical performance. Resistance training biased to eccentric contractions increases serial sarcomere number (SSN) in young muscle, however, maximal eccentric training in old rats previously did not alter SSN and worsened performance. A submaximal eccentric training stimulus may be more conducive to adaptation for aged muscle. The purpose of this study was to assess whether submaximal eccentric training can increase SSN and improve mechanical function in old rats. Twelve 32‐month‐old male F344/BN rats completed 4 weeks of submaximal (60% maximum) eccentric plantar‐flexion training 3 days/week. Pre‐ and post‐training, we assessed in‐vivo maximum isometric torque at a stretched and neutral ankle angle, the passive torque‐angle relationship, and the isotonic torque‐velocity‐power relationship. The soleus and medial gastrocnemius (MG) were harvested for SSN measurements via laser diffraction, with the untrained leg as a control. SSN increased 11% and 8% in the soleus and MG, respectively. Training also shifted optimal torque production towards longer muscle lengths, reduced passive torque 42%, and increased peak isotonic power 23%. Submaximal eccentric training was beneficial for aged muscle adaptations, increasing SSN, reducing muscle passive tension, and improving dynamic contractile performance.

## 1 INTRODUCTION

Muscle fascicle length (FL) becomes shorter with age driven by a loss of in‐series aligned sarcomeres (Hinks et al., 2024; Hinks, Hawke, et al., 2023; Hooper, 1981; Power et al., 2021). This loss of serial sarcomere number (SSN) contributes to muscle atrophy and impaired mechanical function (e.g., reduced muscle power production and elevated passive tension) in old age (Hinks, Hawke, et al., 2023; Narici et al., 2016). We recently showed aged muscle retains the ability to re‐add sarcomeres in series following disuse atrophy, and SSN was notably more adaptable than measures of parallel muscle morphology (i.e., muscle thickness and physiological cross‐sectional area (PCSA)) (Hinks & Power, 2024). Hence, increasing FL by adding sarcomeres in series represents an appealing target for training interventions to improve performance in older adults.

The most common exercise intervention to stimulate sarcomerogenesis is resistance training biased to active lengthening (eccentric) contractions (Franchi et al., 2017; Hinks, Franchi, & Power, 2022). In young healthy rats, eccentric training can increase SSN by up to 8% (Butterfield et al., 2005; Hinks et al., 2024; Hinks, Jacob, et al., 2022; Lynn et al., 1998), and in young adult (age ~ 25 years) humans SSN adaptations have been assumed via increases in FL as measured by ultrasound (Blazevich et al., 2007; Franchi et al., 2014). Increases in FL following submaximal (60%–80% maximum) eccentric training have also been reported in older adults (age ~ 70 years) (Quinlan et al., 2021; Reeves et al., 2004, 2009), however, assuming sarcomerogenesis from ultrasound‐derived FL holds limitations without simultaneous measurement of sarcomere length (SL) (Hinks, Franchi, & Power, 2023). Direct measurement of SL in humans is invasive with limited accessibility (Pincheira et al., 2021), therefore, animal models are needed to investigate the ability for eccentric training to induce sarcomerogenesis in aged muscle.

We recently showed maximal eccentric training induced sarcomerogenesis and beneficial mechanical adaptations in young rats, however, sarcomerogenesis was blunted and function was worsened in old rats (Hinks et al., 2024), likely due to an age‐related impaired recovery from muscle damage following high‐intensity eccentric exercise (McBride et al., 1995). Therefore, the purpose here was to investigate if aged muscle can add sarcomeres in series and improve mechanical function following submaximal eccentric training. We hypothesized that submaximal eccentric training would induce serial sarcomerogenesis in aged muscle and owing to the beneficial mechanical adaptations of longitudinal muscle growth (Hinks, Franchi, & Power, 2022) would correspond to a broadening of the torque‐angle relationship and improvements in power.

## 2 METHODS

### 2.1 Animals

Twelve 32‐month‐old male Fisher 344/Brown Norway rats were obtained (Charles River Laboratories, QC, Canada). The University of Guelph's Animal Care Committee (AUP #4905) approved all protocols. Rats were housed at 23°C in groups of two or three and given ad‐libitum access to a Teklad global 18% protein rodent diet (Envigo, Huntington, Cambs., UK) and room‐temperature water. Pre‐training mechanical testing was completed at 32 months of age then training commenced 4–6 days later. Training lasted 4 weeks, then post‐training mechanical testing was completed 72 h following the final training session. The soleus and medial gastrocnemius (MG) were dissected for SSN determinations. The left leg completed training while the right leg acted as an internal control in accordance with previous studies (Hinks et al., 2024; Hinks, Franchi, & Power, 2023; Kinney et al., 2017; Williams & Goldspink, 1978).

### 2.2 Data acquisition during mechanical testing and training

A 701C High‐Powered, Bi‐Phase Stimulator (Aurora Scientific, ON, Canada) was used to evoke transcutaneous muscle stimulation as described previously (Hinks et al., 2024). Torque, angle, and stimulus trigger data were sampled at 1000 Hz with a 605A Dynamic Muscle Data Acquisition and Analysis System (Aurora Scientific, ON, Canada).

### 2.3 Mechanical testing

As described previously (Hinks et al., 2024), rats were anesthetized with isoflurane and positioned supine on a heated platform (37°C). The left leg was shaved and fixed to a force transducer/length controller foot pedal via tape, with the knee immobilized at 90°. Each mechanical testing session began with determination of the stimulation current for maximal active plantar flexion torque (frequency = 100 Hz, pulse duration = 0.1 ms, train duration = 500 ms) at an ankle angle of 90° (full plantar flexion = 180°), which was the current used throughout the remainder of the testing session. A 100 Hz stimulation was then completed at an ankle angle of 70°. Active torque was measured by subtracting the minimum value at baseline (passive torque) from the total torque during stimulation (Chen et al., 2020; Hinks, Jacob, et al., 2022). A passive torque‐angle relationship was then constructed by recording the minimum passive torque following 5 s of stress‐relaxation at ankle angles of 100, 95, 90, 85, 80, 75, and 70°. A torque‐angular velocity‐power relationship was then constructed from isotonic contractions. For each isotonic contraction, the ankle started at 70°, then contracted against a load clamp to a maximum angle of 110°. Isotonic (i.e., constant torque) contractions were performed at load clamps equating to 10%, 20%, 30%, 40%, 50%, 60%, 70%, and 80% of the maximum isometric torque at 70°, in a randomized order. Angular velocity was recorded as the maximum time derivative of the angular displacement during the isotonic contraction (Hinks et al., 2024). Two minutes of rest separated each stimulation to minimize the development of muscle fatigue.

The estimated maximum shortening velocity at zero load (V_max_), maximum power (torque multiplied by angular velocity), and torque and velocity at peak power were determined by fitting the measured torque and angular velocity values to a rectangular hyperbolic curve (Hill & Sec, 1938):

$$V=\frac{\left(F_{\max}+a\right)b}{F+a}-b$$

with F_max_ being maximum isometric torque at 70°, *F* and *V* representing the isotonic load clamp's torque and angular velocity, respectively, and *a* and *b* representing Hill's thermodynamic constants with the units of torque (N·m) and velocity (°/s), respectively. All curve‐fitting was performed in Python 3 using least square error optimization.

### 2.4 Isokinetic eccentric training

The submaximal eccentric training protocol was modified from our previous maximal protocol (Hinks et al., 2024). Training lasted 4 weeks and occurred 3 days/week (Monday, Wednesday, Friday). Throughout training, we used a stimulation current that corresponded to 60% of the maximum isometric torque.

At the start of each session, the stimulus current was first adjusted to produce maximum isometric torque (pulse duration = 0.1 ms, frequency = 100 Hz, train duration = 500 ms) at an ankle angle of 90°. The current was then adjusted to produce an isometric torque that as closely as possible matched 60% of maximum isometric torque (Figure S1). Maximum isometric torque was recorded for each training session to track changes in muscle strength throughout the training period.

Each eccentric repetition consisted of three phases: (1) a 500‐ms pre‐activation at 110°; (2) active lengthening to 70°; and (3) 3 s of deactivation followed by a return to 110° at 20°/s. An additional 3 s of rest were provided before the next repetition. To progress the eccentric training stimulus throughout, we increased the number of repetitions and the velocity of the eccentric contractions, in accordance with our previous training study (Hinks et al., 2024). During Week 1, rats completed 3 sets of 8 repetitions at 40°/s. In Week 2, they completed 3 sets of 9 repetitions at 40°/s. Weeks 3 and 4 consisted of 3 sets of 9 and 10 repetitions, respectively, at 80°/s. Two minutes of rest were provided between each set.

### 2.5 Serial sarcomere number determinations

Following post‐training mechanical testing, rats were euthanized via isoflurane anesthetization followed by CO_2_ asphyxiation and cervical dislocation. The hindlimbs were amputated and fixed in 10% phosphate‐buffered formalin with the ankle and knee pinned at 90°. After fixation for 1–2 weeks, the muscles were dissected and rinsed with phosphate‐buffered saline. The muscles were then digested in 30% nitric acid for 6–8 h to remove connective tissue and allow for individual muscle fascicles to be teased out (Butterfield et al., 2005; Hinks, Jacob, et al., 2022). Experimenters were not blinded to the control and experimental leg to ensure consistency in transferring muscles between these processing steps.

For each muscle, two fascicles were obtained from each of the proximal, middle, and distal regions and averaged for the reporting of data. Dissected fascicles were placed on glass microslides then FLs were measured using ImageJ (version 1.53 k, National Institutes of Health, USA) from pictures captured by a level, tripod‐mounted digital camera, calibrated to a ruler in plane with the fascicles. SL measurements were taken at six locations proximal to distal along each fascicle via laser diffraction (Coherent, Santa Clara, CA, USA) with a 5‐mW diode laser (~1 mm beam diameter, 635 nm wavelength) and custom LabVIEW program (Version 2011, National Instruments, Austin, TX, USA) (Lieber et al., 1984). For each fascicle, the six SL measurements were averaged to determine average SL. Given the laser diameter of ~1 mm, one SL measurement itself represents an average of 100s–1000s of SLs. Our total quantity of SL and FL measurements is consistent with previous studies (Butterfield et al., 2005; Chen et al., 2020; Hinks, Jacob, et al., 2022). SSN of each fascicle was calculated as:

$$\begin{array}{c} \text{Serial sarcomere number}\\ =\text{fascicle length}/\text{average sarcomere length}. \end{array}$$

### 2.6 Determination of physiological cross‐sectional area

To gain further insight on changes in muscle contractile tissue in parallel, we calculated PCSA (in cm^2^) using the equation (Chen et al., 2020; Ward & Lieber, 2005):

$$\text{PCSA}=\frac{\text{Muscle mass}\;}{\text{Muscle density}\times \text{normalized}\;\mathrm{FL}}$$

Muscle density was assumed to be 1.112 g/cm^3^ (Ward & Lieber, 2005). Normalized FL was calculated using FL of dissected fascicles and measured SL in the equation (Ward & Lieber, 2005):

$$\text{Normalized}\;\mathrm{FL}=\mathrm{FL}\;\frac{\mathrm{SLo}}{\text{measured}\;\mathrm{SL}}$$

SLo represents optimal SL of rat muscle at rest, assumed to be ~2.7 μm based on previous literature (Chen et al., 2020; Zuurbier et al., 1995). Maximum isometric torque normalized to the combined PCSA of the soleus and MG was also recorded, with post‐training torque normalized to trained muscle PCSA, and pre‐training torque normalized to untrained muscle PCSA.

### 2.7 Statistical analysis

In SPSS Statistics Premium 28, normality of data was confirmed using Shapiro–Wilk tests. Differences in SSN, SL, FL, muscle wet weight, and PCSA between the trained and untrained leg were assessed by one‐way analysis of variance (ANOVA). Changes in V_max_, peak power, and torque and velocity at peak power from pre‐ to post‐training were also assessed via one‐way ANOVA. Two‐way ANOVAs assessed training‐induced changes in absolute (mN·m) and normalized (to PCSA; mN·m/cm^2^) maximum isometric torque (training [pre, post] × angle [90°, 70°]), passive torque (training [pre, post] × angle [100°–70°]), and angular shortening velocity (training [pre, post] × load [10%–80%]). Two‐way ANOVA was used in these cases to allow us to investigate differences in the overall shapes of the active and passive torque‐angle relationships and torque‐velocity relationship. A Greenhouse–Geisser correction for sphericity was applied for all ANOVAs, and a Sidak correction was applied to all pairwise comparisons. Significance was set at *α* = 0.05.

## 3 RESULTS

### 3.1 Data exclusion

One rat died between the penultimate and final training sessions and was excluded from all analyses (now *n* = 11). One additional rat was excluded from analyses of mechanical data due to technical issues with its experimental setup at post‐training testing. Thus, the final sample size was *n* = 11 for morphological data and *n* = 10 for mechanical data.

### 3.2 Training‐induced changes in muscle morphology

**FIGURE 1:**
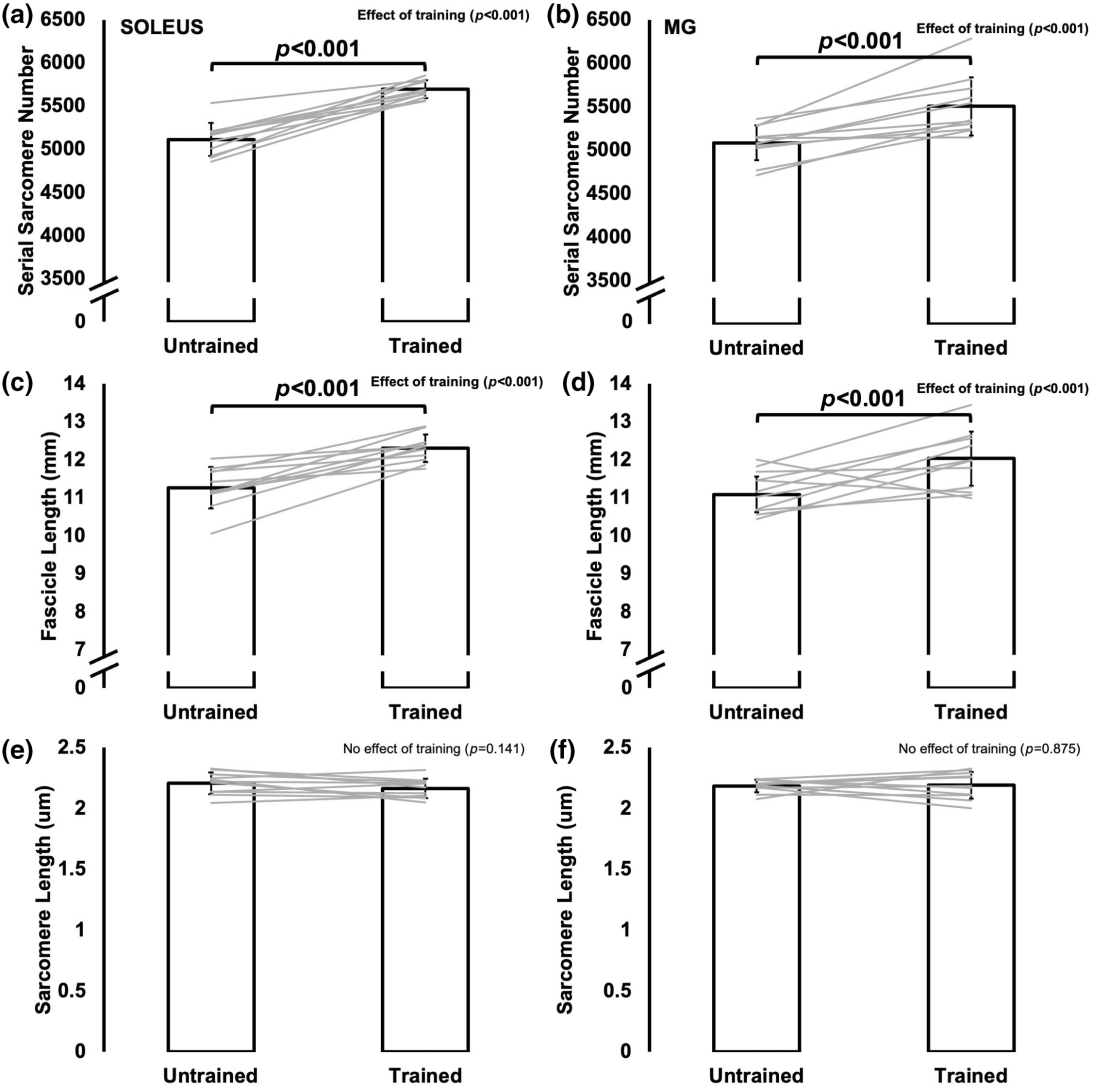
Differences in soleus and medial gastrocnemius (MG) serial sarcomere number (a, b), fascicle length (c, d), and sarcomere length (e, f) between the untrained and trained leg (*n* = 11). Data are mean ± standard deviation. Significance was *p* < 0.05.

SSN increased 11% in the soleus (*p* < 0.001) and 8% in the MG (*p* < 0.001) from the untrained to trained leg (Figure 1a,b). SL as measured at 90° did not differ between legs (soleus: *p* = 0.141; MG: *p* = 0.875) (Figure 1e,f), therefore these increases in SSN resulted in 9% longer FLs as measured at 90° in both muscles (soleus: *p* < 0.001; MG: *p* < 0.001) (Figure 1c,d).

**FIGURE 2:**
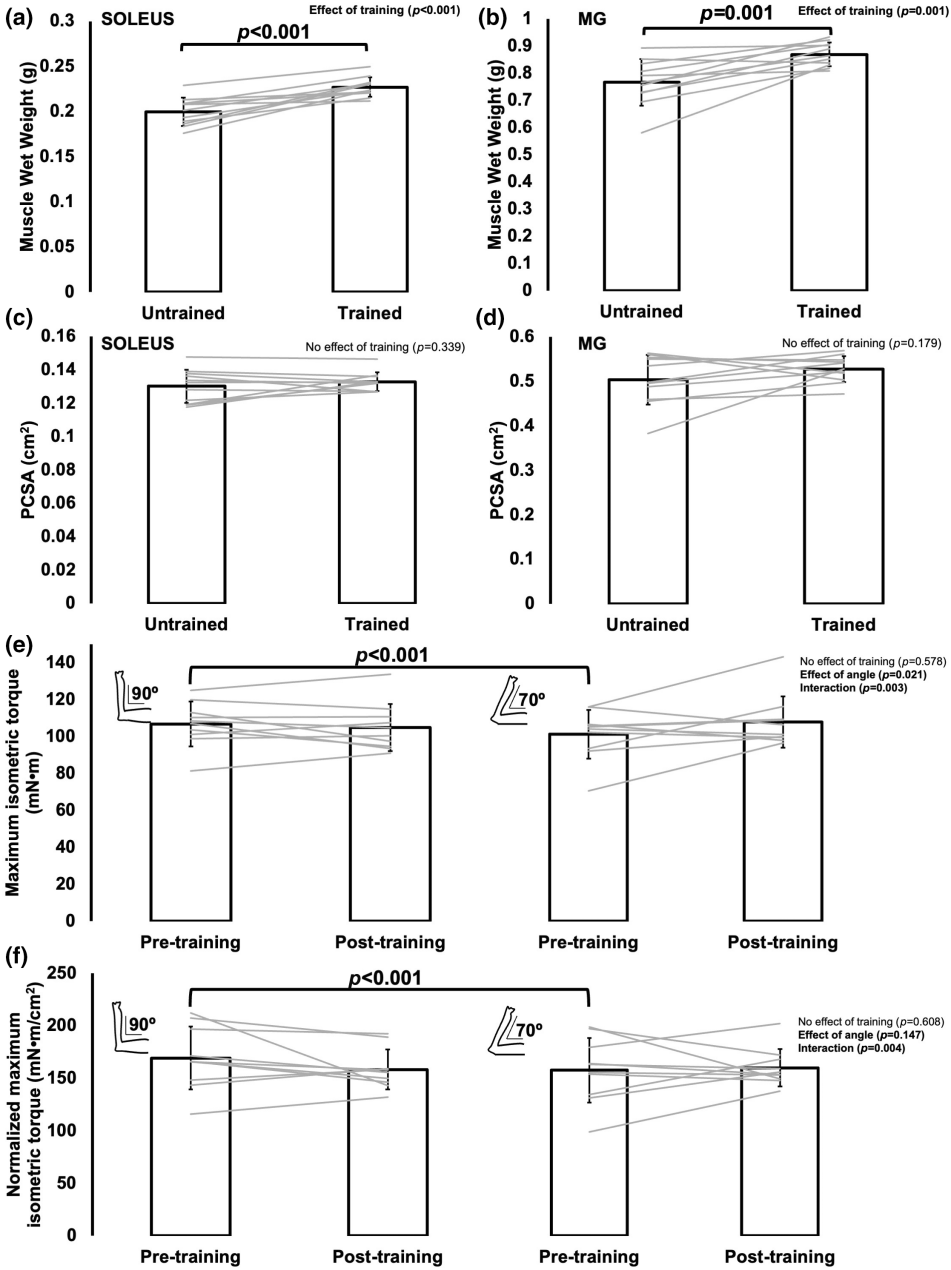
(a–d): Differences in soleus and medial gastrocnemius (MG) muscle wet weight (a, b) and physiological cross‐sectional area (PCSA) (c, d) between the untrained and trained leg (*n* = 11). (e, f): Training‐induced changes in absolute (e) and normalized (f) maximum isometric torque at ankle angles of 90° and 70° (*n* = 10). Data are mean ± standard deviation. Significance was *p* < 0.05.

Muscle wet weight also increased 14% (*p* < 0.001) and 13% (*p* = 0.001) from the untrained to trained leg for the soleus and MG, respectively (Figure 2a,b). This increase in muscle wet weight seemed mostly attributed to contractile tissue added in series as PCSA did not differ between the untrained and trained leg for the soleus (*p* = 0.339) or MG (*p* = 0.179) (Figure 2c,d).

### 3.3 Training‐induced changes in muscle mechanical performance

For both absolute and normalized (to PCSA) maximum isometric torque, there was a training×angle interaction (absolute: *p* = 0.003; normalized: *p* = 0.004). Neither torque at 90° (absolute: *p* = 0.448; normalized: *p* = 0.196) nor 70° (absolute: *p* = 0.178; normalized: *p* = 0.815) changed from pre‐ to post‐training (Figure 2e,f). However, pre‐training, torque at 90° was greater than at 70° (absolute: *p* < 0.001; normalized: *p* < 0.001), whereas post‐training torque no longer differed between these angles (absolute: *p* = 0.126; normalized: *p* = 0.136) with torque at 70° being on average greater (Figure 2e,f). This lack of difference in torque between a neutral and stretched angle post‐training indicates a training‐induced shift in the torque‐angle relationship such that a broader range of angles were close to optimal torque production throughout the joint range of motion.

**FIGURE 3:**
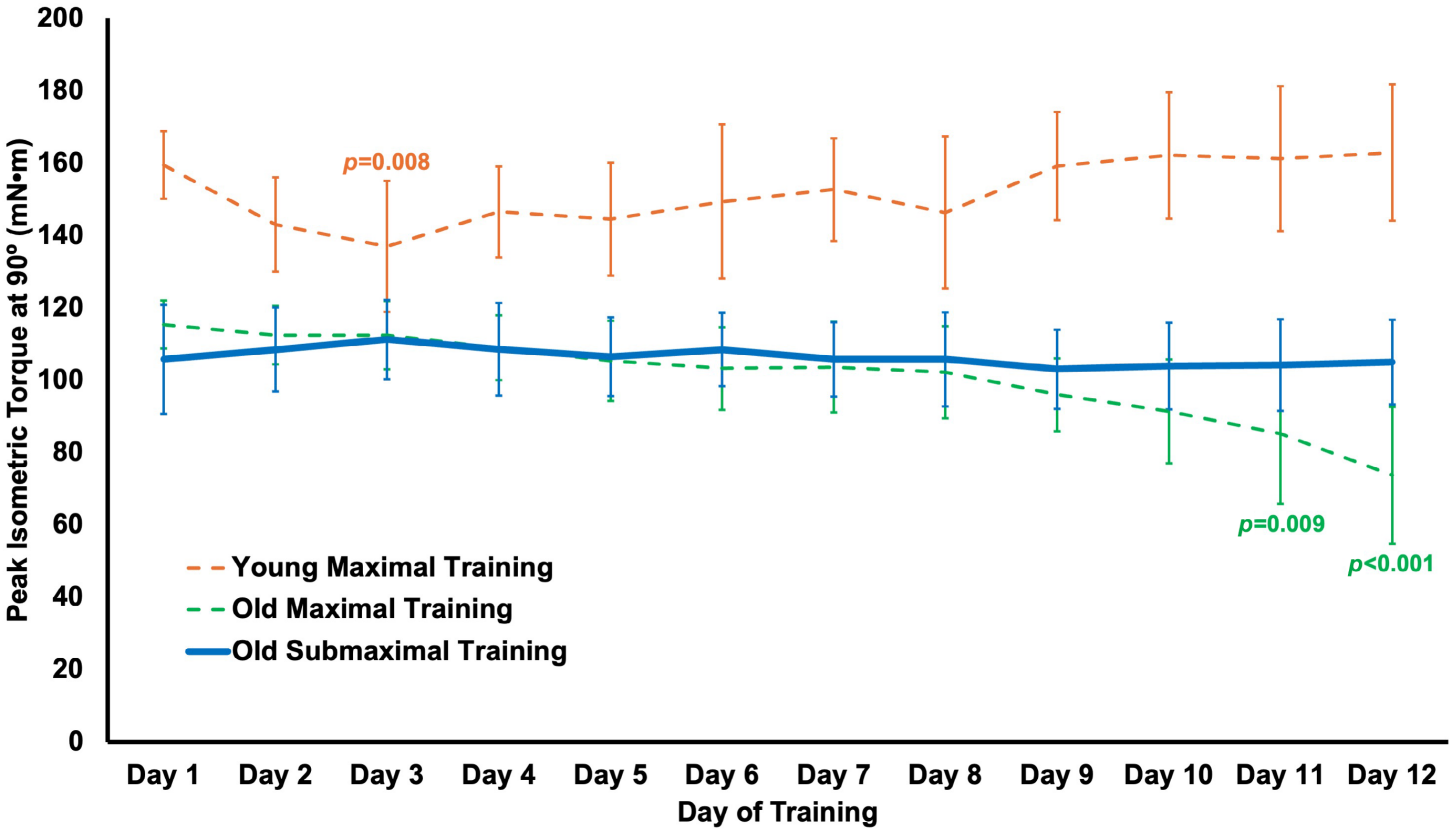
Changes in maximum isometric torque at an ankle angle of 90° throughout the training period in the present study (solid line, old submaximal eccentric training; *n* = 10) plotted alongside our previous study's data (reference (Hinks et al., 2024) for maximal eccentric training (dashed lines) in young (*n* = 10) and old rats (*n* = 11). Data are mean ± standard deviation. Significant difference from baseline was *p* < 0.05.

Torque at 90° also did not change throughout the training period (no effect of training day, *p* = 0.156) unlike our previous maximum eccentric training study in old rats, in which torque at 90° progressively decreased (Hinks et al., 2024) (Figure 3).

**FIGURE 4:**
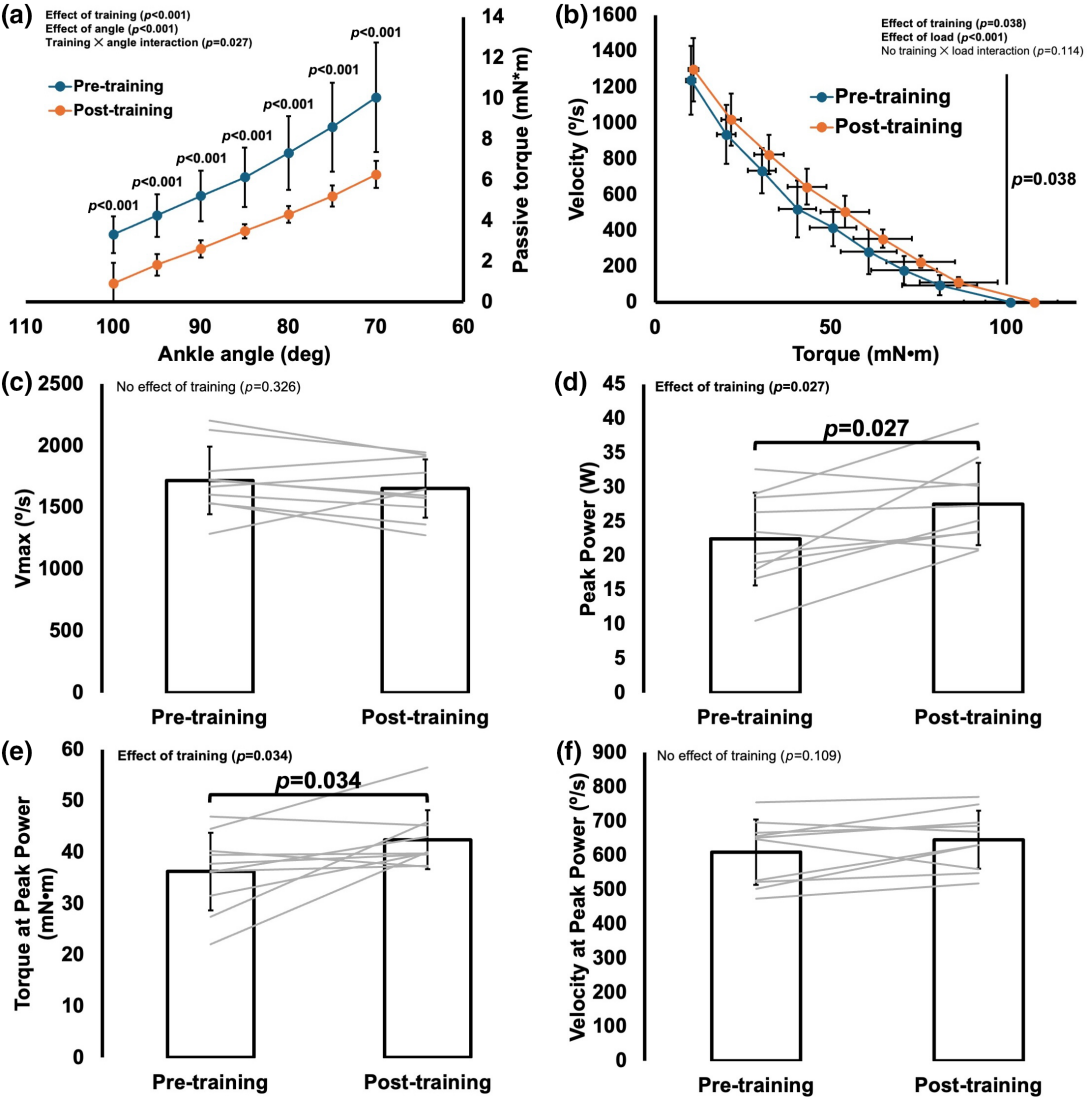
Training‐induced changes in the passive torque‐angle relationship (a), the torque‐angular velocity relationship (b), maximum shortening velocity (V_max_) (c), peak power (d), torque at peak power (e), and velocity at peak power (f) (*n* = 10). Data are mean ± standard deviation. Vertical line in B represents an effect of training. Significance was *p* < 0.05.

For passive torque, there was a training×angle interaction (*p* = 0.027). Both pre‐ and post‐training, passive torque increased continuously as the muscles stretched from an ankle angle of 100°–70° (all comparisons *p* < 0.001). More importantly, there was a training‐induced 38% to 72% reduction (all *p* < 0.001) in passive torque across all joint angles (Figure 4a).

For angular shortening velocity, there was the expected effect of load such that velocity decreased with increasing load (*p* < 0.001). There was also an effect of training (*p* = 0.038) but no interaction (*p* = 0.114), with angular velocity increasing 13% across all loads combined from pre‐ to post‐training (Figure 4b). However, training did not affect V_max_ (*p* = 0.326) (Figure 4c).

Peak power also showed an effect of training, increasing 23% (*p* = 0.027) (Figure 4d). This training‐induced increase in peak power was driven by increased torque rather than velocity, as torque at peak power increased 17% (*p* = 0.034) (Figure 4e) while velocity at peak power did not change (*p* = 0.109) (Figure 4f).

## 4 DISCUSSION

Previously we showed maximal eccentric training induced serial sarcomerogenesis in young rats, but in old rats did not change SSN and further worsened muscle mechanical function (Hinks et al., 2024). Since eccentric contraction‐induced muscle damage is more prevalent with increasing contraction intensity (Chen et al., 2007), and old rats take longer to recover from damage (McBride et al., 1995), sarcomerogenesis was likely blunted due to accumulation of muscle damage and insufficient recovery between each training session. The present study aimed to determine whether a submaximal (60% of maximum torque) eccentric training stimulus would better increase SSN and improve mechanical function in old rats, as suggested by studies on humans using loads equivalent to 50%–80% of 1‐repetition maximum (Baxter et al., 2024; Quinlan et al., 2021; Reeves et al., 2004, 2009).

Indeed, we observed an 11% and 8% increase in SSN of the soleus and MG, respectively, following 4 weeks of submaximal isokinetic eccentric training. These increases in SSN manifested as 9% longer FLs, as resting SLs were unchanged (Figure 1). The increased SSN likely also contributed to the 13%–14% greater muscle wet weights. Given there was no difference in PCSA between untrained and trained muscles and no training‐induced change in maximum isometric torque, addition of sarcomeres in parallel was likely minimal. Our findings are consistent with previous studies that observed increased SSN following eccentric training in young rats (Butterfield et al., 2005; Chen et al., 2020; Hinks et al., 2024; Hinks, Jacob, et al., 2022; Lynn et al., 1998). Importantly, we now show that training‐induced sarcomerogenesis is also possible in old rats.

In contrast to our previous maximal eccentric training study (Hinks et al., 2024), we did not observe a progressive decrease in maximum isometric torque throughout the submaximal training period, and instead observed no change in torque (Figure 3). This comparison further indicates that maximal eccentric training (Hinks et al., 2024) induced dysfunctional remodeling leading to impaired muscle mechanical performance, while submaximal eccentric training was a low enough intensity to allow sufficient recovery and adaptation between sessions.

We did not observe any training‐induced increases in maximum isometric torque or shortening velocity. However, and perhaps more insightful into the unique adaptations to longitudinal muscle growth, we observed a broadening of the torque‐angle relationship such that torque was closer to optimal throughout the joint range of motion, as indicated by torque being greater at an ankle angle of 90° (neutral) than 70° (stretched) pre‐training, but no longer different between these angles post‐training. A rightward‐shifted, broadened force‐length curve has been observed alongside increased SSN previously (Butterfield & Herzog, 2006; Hinks, Jacob, et al., 2022; Williams & Goldspink, 1978), and is likely associated with a reestablishment of optimal SL at a longer muscle length due to increased SSN.

Broadening of the torque‐angle relationship likely also contributed to the training‐induced 23% increase in peak isotonic power (Figure 4d) by making torque closer to optimal throughout the joint range of motion (Akagi et al., 2020) and increasing the force component of power. This effect on the force component of power seems especially likely given torque at peak power increased 17% (Figure 4e). As angular velocity at peak power remained unchanged (Figure 4f), the velocity component of power contributed less to the improved peak power despite a modest effect of training on angular shortening velocity across all loads (Figure 4b).

Submaximal eccentric training also induced a 38%–72% reduction in passive torque (Figure 4a). Decreased muscle passive force (Hinks, Jacob, et al., 2022) and joint passive torque (De Jaeger et al., 2015) have been observed alongside increased SSN previously, and could be due to shorter SLs at a given absolute muscle length, reducing passive force contributed by sarcomeric structures (e.g., titin) (Hinks, Franchi, & Power, 2022). The reduction in passive torque could have also resulted from remodeling of the extracellular matrix, which can occur following eccentric exercise (Hyldahl et al., 2015; Mackey et al., 2011), but those measures were beyond the scope of this study. Considering resting SL at an ankle angle of 90° did not differ between untrained and trained muscles, extracellular matrix remodeling likely accounted for most of the observed reduction in passive torque.

## 5 CONCLUSION

We found that eccentric training at 60% maximum induced sarcomerogenesis in the old rat plantar flexors, suggesting that “less is more” when designing training programs for longitudinal growth of aged muscle. Concomitantly, there were improvements in muscle mechanical function including reduced passive tension and improved peak isotonic power. The increase in power is especially notable given it is a measure of dynamic muscle performance, applicable to real‐world movement, and a strong predictor of function in older adults.

## AUTHOR CONTRIBUTIONS

A.H., and G.A.P., conceived and designed research; A.H., and E.V., performed experiments; A.H., analyzed data; A.H., E.V., and G.A.P. interpreted results of experiments; A.H., prepared figures; A.H., and G.A.P., drafted manuscript; A.H., E.V., and G.A.P. edited and revised manuscript; A.H., E.V., and G.A.P., approved final version of manuscript.

## FUNDING INFORMATION

This project was supported by the Natural Sciences and Engineering Research Council of Canada (NSERC), grant number RGPIN‐2024‐03782.

## CONFLICT OF INTEREST STATEMENT

No conflicts of interest, financial or otherwise, are declared by the authors.

## ETHICS STATEMENT

Approval was given by the University of Guelph's Animal Care Committee and all protocols followed CCAC guidelines (AUP #4905).

## Supporting information


FIGURE S1:

## Data Availability

All data generated or analyzed during the study are available from the corresponding author upon request.
