# Supplementary material for: Submaximal eccentric resistance training increases serial sarcomere number and improves dynamic muscle performance in old rats
Source: Physiol Rep. 2024 Oct 3;12(19):e70036. doi: 10.14814/phy2.70036 (PMC11449626; doi:10.14814/phy2.70036)
Supplement: Supplementary file 1 — FIGURE S1: [file PHY2-12-e70036-s001.pdf]

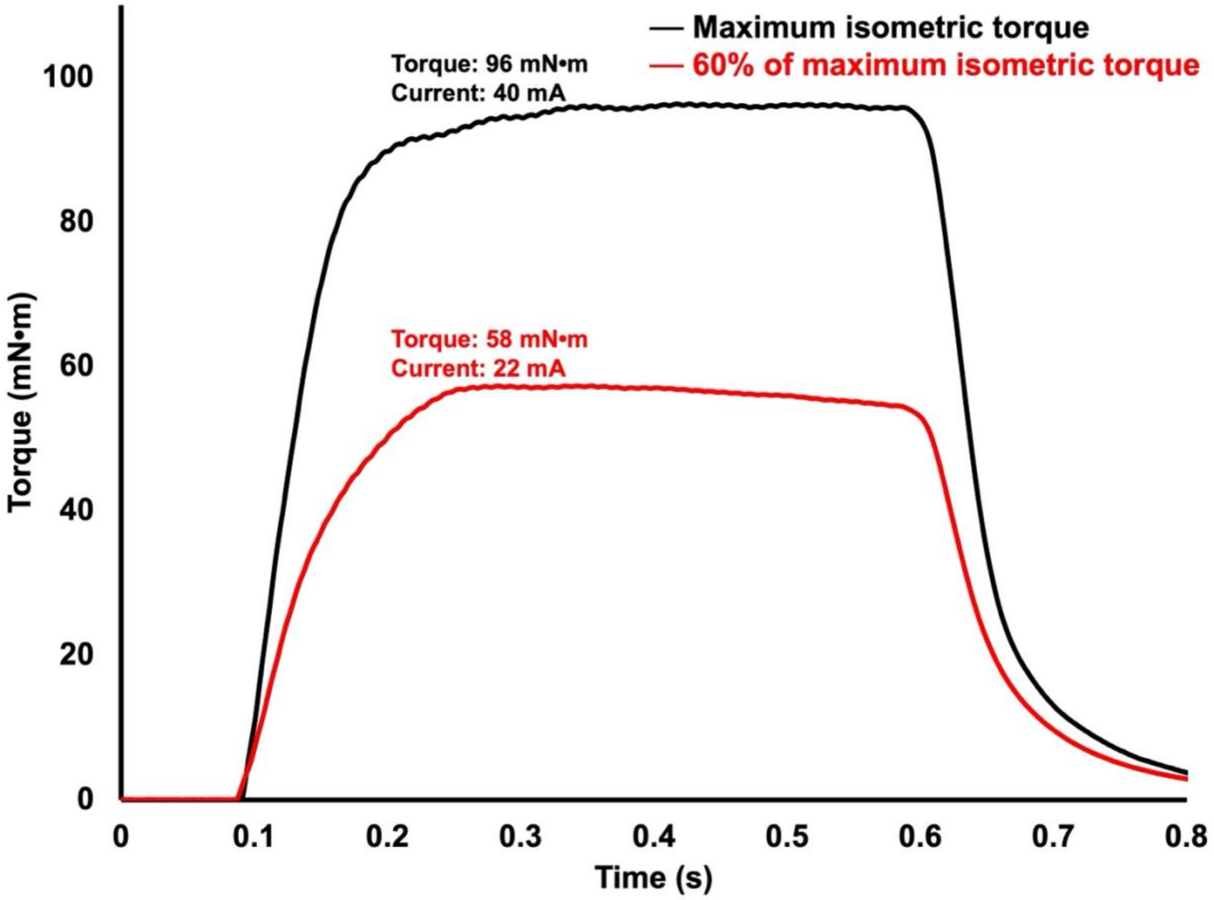

Supplemental Figure S1: Representative raw data traces of a maximum isometric contraction and the corresponding 60% maximum isometric contraction. The stimulation current from the 60% maximum isometric contraction was used for the remainder of that training session during the eccentric contractions.
